# Role of Inflammasomes in Kidney Diseases via Both Canonical and Non-canonical Pathways

**DOI:** 10.3389/fcell.2020.00106

**Published:** 2020-02-27

**Authors:** Huiling Xiang, Feng Zhu, Zhifeng Xu, Jing Xiong

**Affiliations:** ^1^Department of Nephrology, Union Hospital, Tongji Medical College, Huazhong University of Science and Technology, Wuhan, China; ^2^Department of Cardiology, Union Hospital, Tongji Medical College, Huazhong University of Science and Technology, Wuhan, China

**Keywords:** kidney, inflammasome, NLRP3, acute kidney injury, chronic kidney diseases

## Abstract

Inflammasomes, multiprotein complex induced by harmful factors in the body, play a crucial role in innate immunity. Activation of inflammasomes lead to the activation of casepase-1 and then the secretion of inflammatory cytokines, including IL-1β and IL-18, subsequently leading to a type of cell death called pyroptosis. There are two types of signaling pathways involved in the process of inflammasome activation: the canonical and the non-canonical signaling pathway. The canonical signaling pathway is mainly dependent on casepase-1; the non-canonical signal pathway, which was recently discovered, is mainly dependent on caspase-11, but is also meditated by caspase-4, caspase-5, and caspase-8. Kidney inflammation is basically associated with inflammatory factor exudation and inflammatory cell infiltration. Several studies have showed that inflammasomes are closely related to kidney diseases, especially the NOD-, LRR- and pyrin domain-containing 3 (NLRP3) inflammasome, which play a role in regulating kidney inflammation and fibrosis. In this review, we focus on the relationship between inflammasomes and kidney diseases, especially the role of the NLRP3 inflammasome in different kinds of kidney disease via both canonical and non-canonical signal pathways.

## Introduction

Inflammasomes are sensors of innate immunity that are triggered in response to danger signals. They consist kinds of multiprotein complex that include Toll-like receptors (TLRs) and nucleotide-biding oligomerization-domain protein (NOD)-like receptors (NLRs) which trigger inflammation in various diseases especially in immune diseases (Schroder and Tschopp, 2010; Lamkanfi and Dixit, 2012). Inflammasomes have also been related with intestine, lung, neurological diseases and cardiovascular diseases, but how is the inflammasome assembled and even functions in these diseases remains unclear (Opipari and Franchi, 2015; Chen et al., 2016a; Wang et al., 2016; Pinkerton et al., 2017; Lang et al., 2018; Zhang et al., 2020).

The basic structure of an inflammasome consists of a receptor, adaptor, and effector. The receptors are classified based on their structural features and include TLRs, NLRs, Absent in Melanoma 2-Like receptors (ALRs), and pyrin domain (Pyrin) (Sharma and Kanneganti, 2016). The adaptor molecule, apoptosis-associate speck-like protein (ASC), enables the recruitment and activation of caspase-1, which is the effector (Broz and Dixit, 2016). The effector molecule pro-caspase-1 induces maturation of pro-IL-1β and pro-IL-18, causing the cell pyroptosis (Lamkanfi, 2011; Lamkanfi and Dixit, 2014). Innate immunity detects the inflammasome or dangers signals mostly via pattern-recognition receptors (PRRs). There are two kinds of PRRs in innate immune recognition-one recognizes pathogen-associated molecular patterns (PAMPs) and the other danger-associated molecular patterns (DAMPs) (Janeway and Medzhitov, 2002). PAMPs are mostly outer conserved molecules on microbes, while the DAMPs are basically derived from host cells and deliver the signal of injury or cell death (Kanneganti et al., 2007). In the presence of a signal, the PAMPs or DAMPs activate the PRRs on NLRs or TLRs, which triggers the ASC adaptor to activate the effector caspase-1 or caspase-11. The activation of these molecules can lead to the secretion of proinflammatory cytokine pro-IL-1β, pro-IL-18 as well as the cleavage of gasdermin D (GSDMD), thereby triggering cell pyroptosis (Conforti-Andreoni et al., 2011; Kayagaki et al., 2011; Kovacs and Miao, 2017). The canonical pathway involving caspase-1 has been highly researched; however, the role and underlying mechanism of the inflammasomes, particularly involving caspase-11 or other effectors like caspase-4, caspase-5, and caspase-8, which were recently identified to be part of a non-canonical signaling pathway, remains poorly understood (Gurung et al., 2014; Vigano et al., 2015).

Depending on their differences in structure and activation, the inflammasomes are divided into NLRP1, NOD-, LRR- and pyrin domain-containing 3 (NLRP3), NLRC4, IPAF, and AIM2 inflammasomes (de Zoete et al., 2014). Of these, the NLRP3 has been extensively researched and characterized. In this review, we focus on the NLRP3 inflammasome in kidney diseases both in canonical and non-canonical signaling pathway, and provide an update on its roles in kidney disease and discuss their potential therapeutic effects for the future application.

### NLRP3 Inflammasomes and Kidney Diseases

The NLRP3 inflammasome is a member of the NLR families of proteins, which was firstly named in 2002 (Martinon et al., 2002). The NLR families include 23 human genes and 34 mouse genes that can form the inflammasome complex (Tian et al., 2009; Rathinam et al., 2012a). Although NLR genes are expressed in kidney, there is no evidence for a direct link between inflammasome and kidney disease (Vilaysane et al., 2010). NLR family members share the same central NACHT and C-terminal LRR domains, with the different subtypes based on their N-terminus. The common N-terminal domains include acidic transactivation, pyrin, caspase recruitment domain (CARD), and baculoviral inhibitory repeat-like domains (Ting et al., 2008). NLRP3 contains PYD at its D-terminal, which allows its contact with the adapter via PYD-PYD interactions (Sutterwala et al., 2014). Canonical NLRP3 inflammasomes often react with varieties of PRRs, including the danger signal reproduced by itself, bacterial as well as its ligands such as the bacterial DNA, muramyl dipeptide, ATP, and even some virus (Man and Kanneganti, 2015). Non-canonical NLRP3 inflammasomes respond mainly to gram-negative bacteria (Rathinam et al., 2012b).

Although the accurate mechanism of the activation of NLRP3 still remains unknown, some models have been proposed, including the ion fuel model, the reactive oxygen species (ROS) model, and the lysosome rupture model (de Zoete et al., 2014). In ion fuel model, the activation of NLRP3 is majorly meditated by the K^+^ efflux, but also by some other ions like H^+^ or Ca^+^ (Pétrilli et al., 2007; Ichinohe et al., 2010; Lee et al., 2012). The ROS model is based on the fact that ROS from the NADPH oxidase system can accelerate the activation of NLRP3 inflammasome, and inhibiting the ROS can inhibit the activation, although studies have also shown that the activation of NLRP3 in a model of lacking NADPH (Latz, 2010; van Bruggen et al., 2010). Therefore, the role of ROS in the activation of inflammasome requires further thorough investigation. In the lysosome rupture model, the NLRP3 inflammasome is thought to be activated upon sensing the large molecules such as uric acid and cholesterol leading to the lysosome rupture; this reflects the key role of inflammasome in kidney disease and atherosclerosis (Duewell et al., 2010; Gaidt et al., 2017).

Many factors can contribute to the development of kidney diseases, such as sepsis, chemotherapy, contrast agents, infection, and kidney injury that can turn into the end stage of renal disease, following which the only option a patient has is hemodialysis or kidney transplantation (D’Agati and Appel, 1997; Arany and Safirstein, 2003; McCullough, 2008; Chawla et al., 2014; Bellomo et al., 2017). At present, the kidney diseases are mainly diagnosed by renal pathology and most of these diseases have not been thoroughly studied and remain poorly understood. Almost all kidney diseases have the same trait of cytokines expression and infiltration of immune cells, which indicates that the kidney is a target in immune response (Kurts et al., 2013). The *NLRP3* genes are found to express in renal dendritic cells and macrophages, while other non-immune cells seem do not release IL-1β (Martinon et al., 2009; Lichtnekert et al., 2011). Previous study has confirmed that kidney diseases are related to inflammasomes and this association might be direct or indirect (Mulay et al., 2013; Xiong et al., 2015). The activation of inflammasomes in kidney aggravates the damage, while inhibiting certain signaling pathways of inflammasome often alleviates kidney injury. Thus, inflammasome might be a potential target in the treatment of renal disease, suggesting development of novel effective therapeutics for kidney diseases.

### Canonical Signal Pathway

#### Mechanism of Canonical Signal Pathway

The canonical pathway of the activation of inflammasomes is dependent on caspase-1 (Lamkanfi and Dixit, 2014). The adopter protein ASC in inflammasomes possesses a caspase binding site (Sharma and Kanneganti, 2016). Upon any stimuli harmful to the body, the PAMPs or DAMPs activated PRRs trigger the relevant signal pathway as the first protective barrier of the innate immunity (Akira et al., 2006). After detection of risk factors, the ASC recruits the caspase-1, which is cleaved into caspase-1 p10 and caspase-1 p20 subunits (Guo et al., 2015), which meditate the transformation of the proinflammatory cytokines pro-IL-1β and pro-IL-18 into their active forms IL-1β and IL-18 as well as the cleavage of GSDMD. These molecules are related to the NF-kB and MAPK signaling pathways as well as other signaling pathways, which subsequently lead to cell pyrotosis (Fann et al., 2018), and further lead to the damage of organs and dysfunction of the body.

#### Inflammasomes in Acute Kidney Injury

Acute kidney injury (AKI) is a kind of clinical syndrome characterized by a rapid decrease of the kidney function and involves increase of metabolites such as creatinine (Levey and James, 2017). Many factors can result in AKI, such as the sepsis, ischemia reperfusion, chemotherapy and contrast agents (Ozkok and Edelstein, 2014; Gómez and Kellum, 2016; Fähling et al., 2017; Yang et al., 2017). AKI can also occur upon exposure of the body to harmful factors such as the lipopolysaccharide and cisplatin, which activate the innate immunity and inflammasomes (Leemans et al., 2014).

Studies have indicated that the inflammasome plays a role in the AKI model of Ischemia/reperfusion (I/R), cisplatin, sepsis, rhabdomyolysis, and contrast agents (Zhang et al., 2014; Komada et al., 2015; Shen et al., 2016; Nazir et al., 2017). It has already been verified that blocking the NLRP3 inflammasome signal pathway often alleviates the kidney injury in clinical trials (Fan et al., 2019). I/R can directly cause the AKI and the activation of NLRP3 inflammasome (Wen et al., 2018). Recent study showed that mitochondrial reactive oxygen species (mROS)-medicated activation of NLRP3 inflammasomes aggravates the renal injury (Liu et al., 2014). The use of mitochondria target antioxidants Mito TEMPO or the thioredoxin-interacting protein (TXNIP) siRNA can inhibit the production of the NLRP3, which confirmed that the NLRP3 inflammasome could be activated through mROS-TXNIP-NLRP3 signal pathway in I/R injury (Wen et al., 2018).

SIRT family proteins also play a certain role in inflammatory response. SIRT1 reduces ROS production and exerts its anti-inflammatory effect, although whether it can directly regulate inflammation remains controversial (Yeung et al., 2004). In a sepsis AKI model, SIRT3 was shown to have a protective effect on renal mitochondrial injury by reducing the production of ROS, and decreasing the release of IL-1β and IL-18 (Zhao et al., 2016). Overexpressing of SIRT3 promoted the autophagy and reduced the inflammasomes which are assembling in kidney injury caused by sepsis (Zhao et al., 2018). The use of cisplatin is often limited by the presence of AKI during chemotherapy (Huang et al., 2019). Research shows that cisplatin may inhibit autophagy and activate NLRP3 leading to the kidney injury (Qu et al., 2018). However, SIRT3 can protect from the AKI caused by cisplatin by inducing autophagy (Zhao et al., 2018). Moreover, Astragaloside IV can protect from cisplatin-induced kidney injury by inducing the autophagy and suppressing the NF-kB signal pathway, thereby down-regulating the expression of NLRP3 inflammasome (Qu et al., 2019). Moreover, the caspase inhibition can reduce the production of caspase-1 and IL-1β, and thus protect from the cisplatin-induced kidney injury (Lee et al., 2015).

In a model of rhabdomyolysis-induced acute kidney injury (RIAKI), activation and assembly of NLRP3 inflammasomes preceded infiltration of renal immune cells such as macrophages (Komada et al., 2015). Anisodamine shows a protective role in RIAKI by inhibiting the endoplasmic reticulum stress related to thioredoxin-interaction protein (TXNIP)/NLRP3 inflammasome (Yuan et al., 2017). NLRP3 inflammasome could also meditate contrast agent-induced AKI by regulating the cell apoptosis; indeed, NLRP3 inhibition could reduce the rate of apoptosis and the production of ROS in contrast agent-induced AKI (Shen et al., 2016; Tan et al., 2017).

All of these new findings confirmed that the close relationship between acute kidney disease and inflammasome. Though the inflammasome as a therapeutic target has not been widely considered in clinical research, these findings indicate the potential of the inflammasome for developing novel treatment against acute kidney diseases.

#### Inflammasomes in Chronic Kidney Disease

Chronic kidney disease (CKD) is always the end step of many kidney diseases. CKD is divided into five stages based on glomerular filtration rate, and the different stages show different clinical features (Levey et al., 2003). The basic feature of CKD is persistent renal tubular injury and renal fibrosis. CKD has become a huge burden in many countries due to its high morbidity and lack of effective treatment. Recently, many researchers have proved that interfering with the activation of inflammasome could regulate CKD, indicating that the inflammasome is involved in the CKD pathogenesis (Vilaysane et al., 2010). IL-18 and caspase-1 were found to be expressed in renal tubular epithelium as well as in patients with CKD (Matsumoto and Kanmatsuse, 2001; Gauer et al., 2007). In a unilateral ureteral obstruction (UUO) model, caspase-1, IL-1β, and IL-18 showed increased expression, leading to NLRP3 activation, While in *NLRP3* gene knock out mice, less tubular injury and fibrosis were observed after UUO (Vilaysane et al., 2010).

The evaluation of the association between CKD and inflammasome in recent study is mainly focused on the mechanism of mitochondria, aldosterone, proteinuria, and calcinosis (Nishi et al., 2013; Ding et al., 2016; Gong et al., 2016; Anders et al., 2018). Recently, mROS was confirmed related to the CKD, and silencing the *NLRP3* gene could alleviate the associated mitochondrial dysfunction and renal fibrosis (Guo et al., 2017). Aldosterone can directly damage the renal tubule, which is a key point in CKD pathogenesis. Kadoya et al. (2015) showed that high level of aldosterone could activate the inflammasomes via mROS. However, Bi et al. (2018) showed that the Mn(III) tetrakis (4-benzoic acid) porphyrin chloride (MnTABP) can significantly improve the morphology and function of mitochondria and limit the activation of NLRP3 inflammasome, finally reducing the renal injury caused by aldosterone. Proteinuria is an independent risk factor of kidney injury and is considered as an important clinical symptom. A Study showed that proteinuria could function as a DAMP, leading to the renal tubular inflammation via mROS-meditated activation of the NLRP3 inflammasome (Liu et al., 2014).

Calcium carbonate crystal deposition is a crucial factor in the mechanism of tubular injury and fibrosis, which also involves the NLRP3 inflammasome. Anders et al. (2018) showed that calcium oxalate crystal deposition activates the NLRP3 inflammasome via the TGFR signaling pathway, and not through IL-1β, as is commonly thought. Furthermore, it has been shown that NLRP3 can directly promote the TGF-β signal and activation of R-Smad independent of inflammasome (Wang et al., 2013), which offers new insights into the function and potential treatments of NLRP3 inflammasome in kidney disease. In addition, there are other potential treatment or drugs can reduce or inhibit tubular damage by interfering with the signal pathway of NLRP3 inflammasome; these include mitochondria target antioxidants, compound K, Neferine, allopurinol, and ghrelin (Ding et al., 2017; Foresto-Neto et al., 2018; Hsu et al., 2019; Ling et al., 2019).

Though these potential treatments are still under investigation, it will be a promising therapy for CKD in the near future.

#### Inflammasomes in Diabetic Nephropathy

Diabetic nephropathy (DN) is one of the most common microvascular complications in diabetic patients (Flyvbjerg, 2017). It often eventually turns into end stage renal disease, after which the patients can only be sustained through dialysis. In addition, there are still other cardiovascular syndromes that also do not have effective treatments. Although there are many symptomatic treatment measures, the innate mechanism needs to be thoroughly studied and effective treatments need to be urgently developed. Diabetic nephropathy is characterized by typical aseptic inflammation, and the role of inflammasomes in diabetes has also been discovered, as NLRP3 activation was found in diabetic patients as well as podocytes and endothelial cell injury (Boini et al., 2014; Chen et al., 2015; Shahzad et al., 2015; Koka et al., 2019). Studies found that high glucose may be involved in the occurrence of DN by regulating the activation of inflammasome (Chen et al., 2016b; Fu et al., 2017). Another study focus on the mitochondrial ROS-TXNIP-NLRP3 signal pathway to examine the relationship between DN and NLRP3 inflammasome (Feng et al., 2016). The excessive production of mROS can activate the NLRP3 inflammasome through the TRX/TXNIP, but the function of this signal pathway in diabetes pathway remains unknown (Liu et al., 2018). ROS signal pathway has been shown to be closely related to the DN (Qiu and Tang, 2016). Zhou et al. (2018) proved that high glucose can induce the activation of ROS-meditated activation of the NLRP3 inflammasome. Xu et al. (2018) showed that TXNIP can induce the oxidative stress response in glomerular mesangial cells, while Tan et al. (2015) found that TXNIP inhibited tubule-interstitial compartment from acting as an important meditator in the process of tubule-interstitial fibrosis in DN. Moreover, Han et al. (2018) used an antioxidant targeting mtROS (MitoQ) in mice and confirmed that mtROS-TXNIP-NLRP3 inflammasome pathway activation was key for the DN. Apart from these, other potential mechanisms are also under investigation.

Interestingly, Yi et al. (2017) has found that lincRNA-Gm4419 can regulate the inflammasome and tubular fibrosis during DN through the NF-kB/NLRP3 signal pathway. They reported abnormal expression of a number of lncRNAs, including that of Gm4419, in diabetic kidney organization, and showed that the knockdown of lincRNA-Gm4419 could inhibit the inflammasome in kidney (Yi et al., 2017). In another study, it was found that acid ceramidase (AC) deficiency significantly promoted the activation of NLRP3 inflammasome and the secretion of exosomes; the latter further promoted the release of IL-1β in the diabetes model. This study not only confirmed the relationship between inflammasomes and diabetes, but also confirmed the role of exosomes in the development of diabetes (Yuan et al., 2019). This research offers a new perspective on the relationship between inflammasome and kidney disease.

As a potential therapeutic agents against inflammation and DN, Compound K can inhibit the TXNIP/NLRP3 signal pathway and ameliorate the insulin resistance to protect the tubular inflammation (Chen et al., 2016c). Dihydroquercetin also shows a protective role in DN by suppressing the ROS and the NLRP3 inflammasome (Ding et al., 2018). However, further research is required to find and validate effective drugs concerning the role of NLRP3 inflammasome.

#### Inflammasomes in IgA Nephropathy

IgA nephropathy (IGAN) is the most common primary glomerular disease, and is characterized by accumulation or deposition of IgA in kidney (Magistroni et al., 2015). Most such patients eventually show end stage of kidney disease, making it urgent to find effective treatment. As already indicated, the inflammasome has a close relationship with kidney; however, whether it plays a role in the IGAN remains unknown. Recently, Tsai et al. (2017) found that IgA immune complexes can activate the inflammasome through the ROS in macrophage and induce the secretion of IL-1β and caspase-1, which indicated that the NLRP3 might participate in the inflammation in IGAN. Moreover, other factors that regulate NLRP3 activation can relieve the inflammation in IgA to some degree. For example, Antroquinonol can reduce the ROS produced by the IgA-immune complexes-primed macrophages (Yang et al., 2013), while triptolide treatment significantly reduces IL-1β and IL-18 levels and may have anti-inflammatory effects by down-regulating the expression of NLRP3 and TLR4 (He L. et al., 2015). However, there is still a long way to uncover the detailed mechanism of inflammasome in IGAN such as the relationship between IgA-related immune response and inflammasome, offering potential therapeutic approaches.

#### Inflammasomes in Other Kidney Diseases

Hyperhomocysteinemia is an important risk factor for the development of glomerular injury and sclerosis (Zhang et al., 2012). Activation of NLRP3 inflammasome has been shown to be involved in podocyte injury and glomerular sclerosis in hyperhomocysteinemia, and inhibition of NADPH oxidase or knockdown of ASC or caspase-1 inhibition may play a protective effect (Zhang et al., 2012; Abais et al., 2013). These studies confirmed the role of NLRP3 inflammasome in glomerular injury caused by hyperhomocysteinemia. The NADPH oxidase participates in the activation of NLRP3 inflammasome and the downstream H_2_O_2_ is mainly involved (Abais et al., 2014).

Activation of NLRP3 inflammasome is also involved in the development of obesity-related kidney disease. In *ASC* (+/+) mice, high fat diet promoted podocyte damage and the copolymerization of ASC and NLRP3 complex, while in *ASC* (−/−) mice, it could not (Boini et al., 2014). In acid sphingomyelinase (ASM) knockout mice, less caspase-1 and IL-1β production are observed, which suggests that the ASM may participate in the activation of NLRP3 inflammasome during the development of obesity-related kidney disease (Boini et al., 2016).

In addition, NLRP3 inflammasome also plays an important role in crystalline nephropathy. Some crystals, including sodium urate and calcium oxalate, can activate NLRP3 inflammasome (Hutton et al., 2016). Uric acid crystals are engulfed by lysosomes, which leads to lysosome rupture and then activation of NLRP3 inflammasome (Isaka et al., 2016). Blocking the activation of NLRP3 inflammasome, such as blocking IL-1β, may reduce the progression of crystalline nephropathy (Mulay et al., 2013).

### Non-canonical Signal Pathway

#### Mechanism of Non-canonical Pathway

The recently identified non-canonical pathway of the activation of the inflammasome is mainly dependent on caspase-11, and also by caspase-4, caspase-5, and caspase-8. Murine caspase-11, also called caspase-4, is a critical regulatory factor in the activation of caspase-1 in the bacterial infections. Human caspase-4/caspase-5, speculated to be homologous with murine caspase-11, can regulate the release of inflammatory cytokines by binding LPS directly via CARD domains in an LPS stimulated monocytes model (Vigano et al., 2015). Caspase-11, caspase-4, and caspase-5 can all directly bind the LPS, inducing the production of IL-1β (Yang et al., 2015). In the non-canonical pathway, the inflammasomes first assemble due to detection of PAMPs or DAMPs by PRRs, leading to the activation of caspase-11, which in turn has two kinds of effects. One is that the caspase-11 activation leads directly macrophage death, and the other effect is that the caspase-11 functions as a binding partner in activation of caspase-1, leading to the release of the pro-IL-1β and pro-IL-18 and subsequent pyroptosis; that indicates that the caspase-11 can regulate activation of caspase-1 (Kayagaki et al., 2011; Broz et al., 2012; Vigano and Mortellaro, 2013). In a model of septic shock, the caspase-11 was overexpressed in macrophage and it could directly regulate the caspase-1 and caspase-3 (Kang et al., 2002). Another study also found that the LPS-induced caspase-11 in mice or caspase-4 in human can trigger the cleavage of GSDMD and its subsequent insertion into the cell membrane, leading to pyroptosis (Kayagaki et al., 2015). It has also shown that the caspase-11-meditated response to cytoplasmic LPS activates GSDMD, which is essential for pyroptosis and the secretion of IL-1β (He W.T. et al., 2015, Shi et al., 2015). Though the mechanism of GSDMD and its relationship with IL-1β remains to be further investigated, these studies have provided new insights for both canonical and non-canonical signaling pathway in the activation of inflammasome.

Caspase-8 is a kind of initiator caspase that can activate the downstream effectors caspase-3 and caspase-7 (Lavrik and Krammer, 2012). Caspase-8 and its adaptor FAS-associated death domain protein (FADD) can trigger the activation of inflammasome by inducing the release of IL-1β in the absence of caspase-1/caspase-11, thereby indicating the presence of another non-canonical pathway (Bossaller et al., 2012; Gringhuis et al., 2012).

#### Non-canonical Function of NLRP3 Inflammasome in Kidney Disease

Caspase-1, IL-1β, and IL-18 cannot be activated after knockout of NLRP3 inflammasome, suggesting that NLRP3 is essential for the production of these inflammatory components (Shigeoka et al., 2010). Previous studies have confirmed the role of inflammasomes in kidney immunity, supporting the theory that knocking out the inflammasome genes may also alleviate the injury.

In AKI, deficiency of NLRP3 inflammasome provides a protective effect in I/R induced AKI, although blocking IL-1β and IL-18 does not show any protective effect (Iyer et al., 2009; Shigeoka et al., 2010). This implies that NLRP3 inflammasome not only induces the kidney injury by meditating the production of IL-1β and IL-18, but might also be involved in other non-canonical signaling pathway. Knockout of both *NLRP3* and *ASC* showed a protective effect in kidney injury, suggesting that they may function together. Chun et al. confirmed that caspase-8 can form a protein complex with NLRP3 and ASC to regulate apoptosis in a non-canonical manner in epithelial cells (Chung et al., 2016). Interestingly, *NLRP3* knockout showed no protective effects in cisplatin-induced AKI, which further confirms the non-canonical effect of NLRP3 (Kim et al., 2013); however, its role in cisplatin model remains to be further elucidated.

In CKD, increased expression of NLRP3 inflammasome promotes renal epithelial-mesenchymal transformation (EMT) which is related to the phosphorylation of Smad-2 and Smad-3, and this effect is independent of caspase-1, ASC, IL-1β, or IL-18 (Anders and Lech, 2013). The NLRP3 inflammasome has also been identified to affect the TGF-β signal pathway and activate R-Smads independently of the caspase-1, IL-1β, and IL-18 signaling pathway (Wang et al., 2013). In oxalate-induced nephrocalcinosis, *NLRP3* and *ASC* deficient mice failed to develop nephrocalcinosis, suggesting the protective effects of *NLRP3* and *ASC* deficiency (Anders et al., 2018). However, the use of IL-1β inhibitors could not achieve the protective effect, revealing that NLRP3 inflammasome might act on nephrocalcinosis through other non-canonical pathway, possibly involving macrophage polarization and fibrosis (Anders et al., 2018). The molecular mechanism underlying these non-canonical signaling pathways and whether they involve caspase-11, caspase-8, or other unknown pathways requires further investigation, and the existing research progress will also provide a new understanding of the pathogenesis of kidney diseases.

### Conclusion and Future Perspectives

**FIGURE 1:**
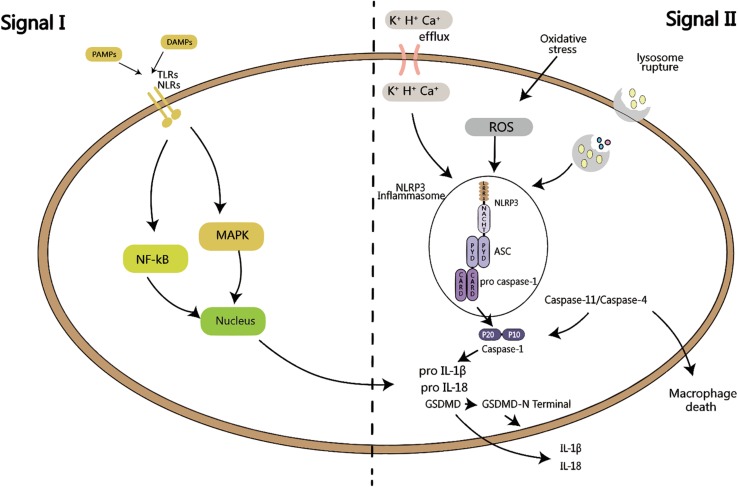
Activation of inflammasome requires two signals: signal I is PAMPs or DAMPs close to the cell, sensed by TLRs or NLRs, and activate MAPK or NF-kB signaling pathway, which in turns produces pro-IL-1β and pro-IL-18. Signal II is K^+^, H^+^ or Ca^+^ efflux, or oxidative stress activation by ROS, or lysosomal rupture, leading to the activation of NLRP3 inflammasome. ASC recruits pro caspase-1, which is then is cleaved into active p20 and p10 fragments, followed by the maturation of IL-1β and IL-18, as well as the cleavage of GSDMD, leading to cell pyroptosis. Both of these signals are called the canonical pathway of inflammation activation. When signal II is mediated by caspase-11/caspase-4, it is called the non-canonical activation pathway of inflammasome.

**FIGURE 2:**
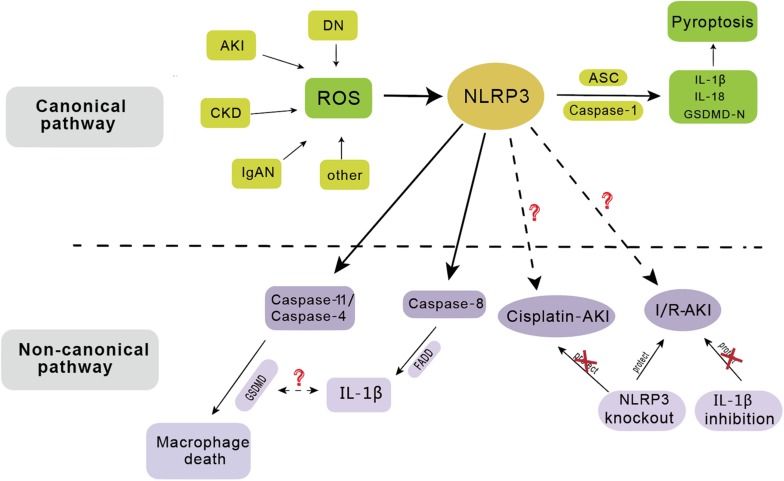
The activation of NLRP3 inflammasome has involved in the pathogenesis of several renal diseases including acute kidney injury (AKI), chronic kidney disease (CKD), diabetic nephropathy (DN), IgA nephropathy (IgAN), etc. In the canonical pathway, NLRP3 functions as a pattern recognition receptor for danger signal. Stimulation with danger or inflammatory signals triggers the formation of a large multimolecular complex, namely, NLRP3 inflammasome, where caspase-1 is activated to cleave its substrates including the precursors of inflammatory cytokine IL-1β to its bioactive form and leads to cell death called pyroptosis. In the non-canonical pathway, NLRP3 inflammasome mainly dependent on caspase-11/caspase-4 or caspase-8, leading to macrophage death. *NLRP3* gene knockout has protective effect on ischemia/reperfusion induced AKI (I/R-AKI), but not on cisplatin induced AKI. IL-1β inhibition has no protective effect on I/R-AKI.

The NLRP3 inflammasome is an intermediate medium for the rapid response of the body to the external injury, including activation of inflammatory cytokine and pyroptotic cell death. The inflammasomes activation by the PRRs and then result in the activation of caspase-1 and then lead to the secretion of IL-1β and IL-18 also with pyroptosis. In the process of NLRP3 activation, both the canonical signal pathway and non-canonical signaling pathways have been shown to play a role in the immune response to the external or internal harmful signals (Figure 1). The relationship of inflammasomes with many diseases, such as lung disease, intestine disease, neurological disease and cardiovascular diseases, has already been tested. In kidney, inflammasomes play a different role in various diseases, and mainly induce the inflammation and dysfunction of kidney; interfering the process of NLRP3 inflammasome activation can regulate the kidney injury. Through the canonical pathway, molecules like SIRT3 can inhibit the kidney injury by decreasing the release of IL-1β and IL-18. In the non-canonical pathway, caspase-8 can form a protein complex with NLRP3 and ASC to regulate apoptosis (Figure 2). Although current studies have confirmed the relationship with NLRP3 inflammasome and kidney disease, the mechanism needs further elucidation. The canonical pathway has been studied extensively, but its clinical applicability to treatment of disease is still ongoing; the non-canonical pathway, which was relatively recently identified, is currently under investigation, and it is hoped that the research will provide insights into mechanisms of kidney disease and its prevention. The existing ways and other unknown mechanisms for non-canonical pathway might be hot topics for future research. In conclusion, a full understanding of the mechanism of inflammasome in kidney disease may help comprehending the pathogenesis of renal disease and solving some of the current clinical problems.

## Author Contributions

HX and FZ wrote the manuscript. ZX contributed to the preparation of this manuscript. JX organized and reviewed the manuscript. All authors have read and approved the final manuscript.

## Conflict of Interest

The authors declare that the research was conducted in the absence of any commercial or financial relationships that could be construed as a potential conflict of interest.
